# Use of the transposition u-shaped palatal flap and polypropylene mesh to correct oronasal communication after resection of maxillary osteosarcoma in a dog

**DOI:** 10.29374/2527-2179.bjvm004824

**Published:** 2024-11-18

**Authors:** Fabiano Luiz Dulce de Oliveira, Sylvia Cristina Silva de Azevedo, Valesca Oliveira de Sousa, Carlos Henrique Teles dos Santos, Maria Eduarda Pereira Coelho, Stéfany Freitas Teixeira Silva, Ticiana do Nascimento França

**Affiliations:** 1 Veterinarian, MSc. Programa de Pós-Graduação em Medicina Veterinária (PPGMV), Departamento de Medicina e Cirurgia Veterinária (DMCV), Instituto de Veterinária (IV), Universidade Federal Rural do Rio de Janeiro (UFRRJ). Seropédica, RJ, Brazil.; 2 Veterinarian, MSc. Escola de Policlínica Veterinária, (UNIFAA). Valença, RJ, Brazil.; 3 Undergraduate in Veterinary Medicine, UNIFAA. Valença, RJ, Brazil.; 4 Veterinarian, DSc. Departamento de Epidemiologia e Saúde Pública (DESP), IV, UFRRJ. Seropédica, RJ, Brazil.

**Keywords:** axial osteosarcoma, hemimaxillectomy, complication, reconstruction, osteossarcoma axial, hemimaxilectomia, complicação, reconstrução

## Abstract

Although less common in the axial skeleton, osteosarcoma, when present in the facial bones, can generally require invasive surgical procedures, which require large resections followed by reconstructions that can disfigure the patient and predispose them to physiological difficulties. We describe a technique used to correct oronasal communication that occurred after suture dehiscence from a left central hemimaxillectomy procedure in a female Rottweiler diagnosed with osteosarcoma. Clinical and radiographic findings are discussed, as well as the palatal mucosal flap surgical technique associated with the use of a synthetic polypropylene mesh. The patient was monitored for a period of six months, when close monitoring of the healing of the surgical wound was possible with adequate incorporation of the mesh into the local tissue. Although a small part of the flap showed dehiscence, there was no aspiration of food due to the presence of the polypropylene mesh under the affected region. The non-rejection of the implant, good acceptance and good eating capacity of the patient, which resulted in weight gain, must be emphasized. We concluded that the combination of techniques presented here is a technically easy, low-cost and efficient option for the proposed treatment.

## Resumo

Embora menos comum no esqueleto axial, o osteossarcoma, quando presente nos ossos faciais, pode normalmente exigir procedimentos cirúrgicos invasivos, que requerem grandes ressecções seguidas de reconstruções que podem desfigurar o paciente e predispô-lo a dificuldades fisiológicas. Nós descrevemos uma técnica utilizada para corrigir a comunicação oro nasal que ocorreu após deiscência de sutura de um procedimento de hemimaxilectomia central esquerda em uma cadela da raça Rottweiler diagnosticada com osteossarcoma. São discutidos os achados clínicos e radiográficos, além da técnica cirúrgica do retalho de mucosa palatina associada ao uso de tela de polipropileno sintético. A paciente foi monitorada por um período de seis meses, onde foi possível observar frequentemente a cicatrização da ferida cirúrgica, com adequada incorporação da tela ao tecido local. Embora uma pequena parte do retalho tenha apresentado deiscência, não houve aspiração de alimentos, devido à presença da tela de polipropileno sob a região afetada. Vale ressaltar que não houve rejeição do implante, boa aceitação e boa capacidade alimentar do paciente, o que culminou no seu ganho de peso. Conclui-se que a combinação de técnicas aqui apresentadas é uma opção tecnicamente fácil, de baixo custo e que oferece eficiência no tratamento proposto.

## Introduction

Considered as the most common osseous neoplasm in dogs, osteosarcoma is characterized by more frequently affecting long bones of the appendicular skeleton of male animals from big to giant breeds like Boxer, Great Dane, German Shepherd, Irish Setter, Rottweiler, Mastiff, Bernese Mountain Dog, Labrador Retriever, Golden Retriever, Dobermann, Greyhound, and Irish Wolfhound (Serakides & Ocarino, 2023). Regarding the age group, it can affect adult and old animals at an average age of 8.2 years (Culp et al., 2014). Although less common, this tumor is also reported in bones of the axial skeleton, or flat bones, such as in the skull, ribs, vertebrae and pelvis (Albertus, 2011; Farcas et al., 2014; Nelson & Couto, 2023; Sabattini et al., 2017; Straw, 2004). According to Tedardi et al. (2016), about 75% of osteosarcomas affect the appendicular skeleton, and 25% involve the axial skeleton. As for differences in behavior, as in human oral and maxillofacial osteosarcoma, they also seem to be less aggressive than the appendicular osteosarcoma in dogs (Farcas et al., 2014).

The clinical signs in animals with axial skeleton osteosarcoma vary. When located in the maxilla or jawbone region, animals can present with touch-sensitive pain, or pain when they open their mouth, dysphagia, sialorrhea (blood-stained or not), halitosis, local edema with potential eyeball displacement, which may even cause asymmetry or facial disfiguration in more advanced cases (Albertus, 2011; Fossum et al., 2021; Schulz, 2015; Thompson & Dittmer, 2017).

The treatment options vary according to the stage of the tumor. Common treatments include surgery, chemotherapy, radiotherapy and immunotherapy, although a combination of these therapies could be used (Jung et al., 2023). Ideally, local management is achieved with a broad surgical resection resulting in tumor-free margins. Local management failure is the biggest contributor to a poor prognosis. A successful treatment depends on the shape and extension of the neoplasm, as well as the aggressiveness of the therapy. The earlier the diagnosis, the greater the chances of a successful treatment will be (Farcas et al., 2014; Ganguly et al., 2015).

Surgical techniques in the facial region, such as maxillectomy and its variations, typically work to avoid tumor growth and tumor cell dissemination, to support the adjuvant treatment for a better patient prognosis (Fossum et al., 2021). Additionally, when the surgery is efficiently planned, it has a secondary objective, which is to try to reestablish part of the patient’s esthetics and the function of the affected area, which is not always possible. Regardless of the selected technique, the procedure is considered invasive as osteotomies must be performed that may result in intra- and postoperative complications, such as hemorrhage and suture dehiscence, respectively. Therefore, it is up to the surgeon to establish appropriate surgical planning, adequately conduct the technique, and resolve any existing complications (Arzi et al., 2020; Berg, 2012; Radlinsky, 2015).

There are several resources that are mentioned and used to correct wounds in the field of Veterinary Surgery. Specifically, in the field of Dentistry, the main modality for the reconstruction of oronasal communication is the use of mucoperiosteal flaps with various configurations and results (Beckman, 2006; Chambers et al., 2021; Guzu et al., 2021; Peralta & Marretta, 2020; Smith, 2001; Taney et al., 2021; van de Wetering, 2005). In addition to these, muscle flaps (Ciepluch et al., 2023) are also used. The use of synthetic materials (Castejón-González & Reiter, 2023; Edstrom & Smith, 2014) is increasing, especially after the advent of those that can be three-dimensionally printed when associated with advanced imaging (Kim et al., 2018; Liptak et al., 2017).

The purpose of this study is to document an alternative surgical technique to resolve oronasal communication in a dog with maxillary osteosarcoma undergoing a hemimaxillectomy procedure. A transposition U-shaped palatal flap was used over a synthetic polypropylene mesh.

## Case Report

**Figure 1:**
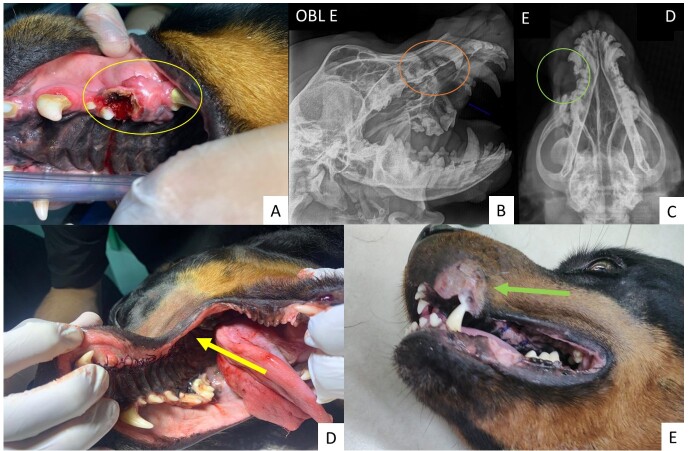
Image sequence of clinical, radiographic, surgical, and postoperative findings in a female, castrated, eight-year-old Rottweiler dog diagnosed with osteosarcoma in the left maxilla. (A) The presence of an amorphous, ulcerated, and infiltrated mass in the vestibular region of the left maxilla bone, tending towards the palatine bone, covering the region from the first to the forth premolars (yellow circle); (B) Left oblique radiographic image of the skull showing decreased radiopacity and distance between the third and fourth upper left premolars with the presence of osteolysis, compatible with the tumor mass region seen in A (orange circle); (C) Ventro-dorsal radiographic image of the skull, showing an increase in volume of soft tissues, compatible with the tumor mass region seen in A (green circle); (D) Postoperative image after the central left hemimaxillectomy procedure, showing the patient’s esthetic aspect after the suture, with a slight depression on the side of the face (yellow arrow); (E) Wound on the lateral face of the left upper lip, compatible with the region where the lower left canine tooth was traumatizing (green arrow). Source: Fabiano Luiz Dulce de Oliveira.

An eight-year-old female Rottweiler was seen at the Polyclinic Veterinary School, University Center of Valença - UNIFAA, with complaints of halitosis (bad breath), which was most likely due to a tumor mass in the maxilla that made it difficult for the dog to eat, even though the animal had appropriate overall body condition and health status. Its owner reported the animal had already been seen by another veterinarian. Even though the owner showed no tests, she stated that the veterinarian suspected it could be an osteosarcoma. After instructions and the owner's authorization, the animal was put under general anesthesia for a better evaluation of its oral cavity, while skull and chest X-rays were taken. During the inspection of the oral cavity, an abnormal growth was seen, which characterized an amorphous ulcerated tumor mass in the left maxilla infiltrating the upper dental arch (Figure 1A).

Both skull X-rays – left oblique and ventro-dorsal – revealed decreased radiopacity, with the presence of osteolysis located in a region that was compatible with that of the mass (Figure 1B and 1C). On the other hand, the left and right, as well as the ventro-dorsal, chest X-rays showed a mild predominantly interstitial pattern, but with no changes that might support metastasis. A biopsy was recommended.

For uncertain reasons, the animal returned only after one month and the oral cavity mass had grown from the vestibular to the palatal region. However, no other changes were seen in the patient’s physical condition, and the owners had no further complaints. The possibility of a partial maxillectomy was discussed for removal of the mass. A blood sample was collected on the same day for preoperative tests (blood count and biochemical analysis), and the following preoperative medications were prescribed: amoxicillin + potassium clavulanate (20 mg/kg / BID / PO for 10 consecutive days), carprofen (2.2 mg/kg/BID/PO for 7 consecutive days), dipyrone (25 mg/kg/TID/PO for 5 consecutive days), and gabapentin (10 mg/kg/BID/PO for 30 consecutive days). The only change found in the blood test was an isolated increase in alkaline phosphatase. Thus, the surgery was scheduled after the owner’s agreement.

A combination of dexmedetomidine (0.003 mg/kg), ketamine (1 mg/kg) and methadone (0.3 mg/kg) were given as a preanesthetic intramuscular injection. The anesthetic induction included the intravenous administration of a combination of fentanyl (0.1 mL/kg) and propofol (5 mg/kg). Isoflurane connected to a 0.8-L/min flow oxygen 100% semi-closed loop was used to maintain the anesthesia. The use of local anesthesia with bupivacaine (5 mg/kg, or 0.25 mL/kg volume) was selected for regional blockade of the maxillary foramen. The animal was positioned on right lateral decubitus to undergo the left central hemimaxillectomy procedure as recommended by Berg (2012). After the gingival incision on the vestibular side, a surgical dissection of the subcutaneous tissue was done using Metzenbaum scissors with all the precautions to prepare the gingival flap to be used later during the synthesis. After complete bone exposure, a partial osteotomy of the left maxillary bones and part of the palatine – from the upper left canine to the fourth upper left premolar – containing a mass was performed using a sagittal blade associated with an oscillating saw under constant irrigation. A 3-cm surgical margin was prioritized in all surrounding tissues. Following the removal of the tumor, there was no local filling, and the previously prepared gingival flap was sutured on the palatine mucosa using a simple interrupted Polyglactin 910 suture pattern (Figure 1D). At the end of the procedure, dipyrone (25 mg/kg), ceftriaxone (25 mg/kg) and meloxicam (0,1 mg/kg) were given intravenously. Stomorgyl® (25 mg/kg / SID / PO for 5 consecutive days), carprofen (2.2 mg/kg / BID / PO for 7 consecutive days), dipyrone (25 mg/kg / BID / PO for 5 consecutive days), tramadol (2 mg/kg/BID/PO for 5 consecutive days), and gabapentin (10 mg/kg/BID/PO for 30 consecutive days) were prescribed to be given at home.

The animal returned for reevaluation after five days. The owner reported that it had not become prostrated at any moment, although the administration of food and medications was rather difficult. The owner also complained that the animal would sneeze and choke quite frequently. During examination, a wound was seen on the lateral face region of the left upper lip, which was compatible with the area touched by the bottom left canine (Figure 1E). The membranes of the ocular and oral mucosa were also noticeably pale, and a new blood sample was taken to get a new blood count. Anemia was confirmed, and supplementation with Eritrós Dog Tabs® (1 tablet/animal PO/SID for 14 consecutive days) was prescribed. The owners were instructed to return with the patient after 14 days to repeat the blood count. As recommended, the owners returned with the dog, and the blood count showed a slight increase in alkaline phosphatase. The owners reported a positive adaptation to the animal’s new condition. Sneezing and choking were still present. The physical examination revealed that the animal was maintaining its physical condition, was active and showed an improvement in the color of the mucosa. However, an oronasal communication was detected in the surgery region due to partial suture dehiscence. At this point, a new surgery was proposed to repair the defect, where a gingival flap was suggested with or without the use of a synthetic material to be determined during the surgery.

**Figure 2:**
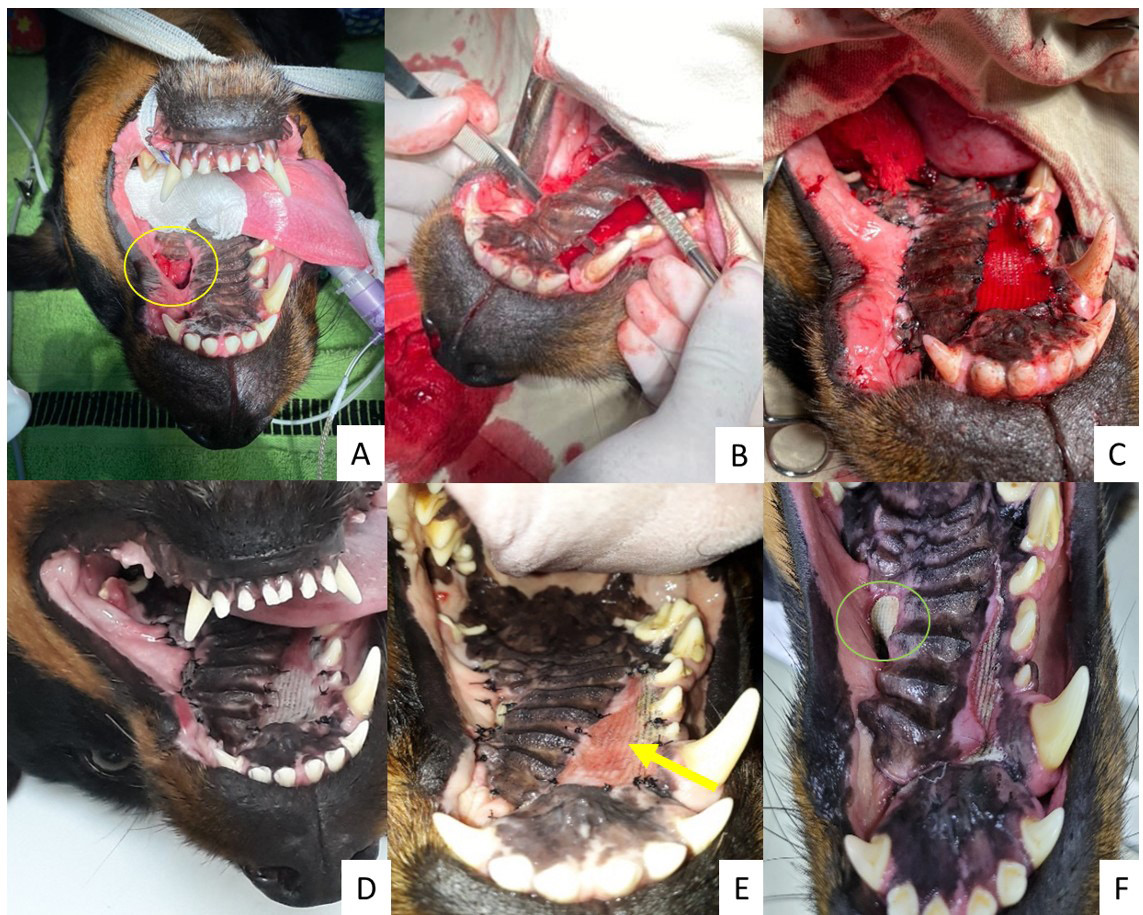
Intra- and postoperative image sequence of the oral cavity of a female, castrated, eight-year-old Rottweiler dog diagnosed with osteosarcoma in the left maxilla, undergoing a correction surgery of the oronasal communication with the use of a palatal mucosal flap combined with a polypropylene mesh. (A) Oronasal communication to be corrected due to suture dehiscence after the central hemimaxillectomy procedure (yellow cicle); (B) Preparation of the palatal mucosal flap; (C) Immediate result of the palatal mucosal flap combined with the use of a polypropylene mesh after the surgery; (D) Palatal mucosal flap combined with the use of a polypropylene mesh during the first return visit after 14 days, demonstrating suitable positioning of the flap and polypropylene mesh with the absence of oronasal communication; (E) Palatal mucosal flap combined with the use of a polypropylene mesh before removal of the stitches after 24 days, demonstrating adequate positioning of the flap and mesh with the absence of oronasal communication (Initial granulation tissue interwoven in the mesh orifices - yellow arrow); (F) Palatal mucosal flap combined with the use of a polypropylene mesh after a six-month period, demonstrating appropriate positioning of the flap and mesh with almost complete incorporation of the mesh into the mucosa. (Presence of an orifice where the third premolar should be protected by the mesh - green circle). Source: Fabiano Luiz Dulce de Oliveira.

Due to the availability of qualified professionals and material to perform this technique, the surgery was scheduled to take place 10 days later. On the day of the surgical intervention, the animal was pre-medicated and anesthetized by following the same protocol as the previous procedure. The animal was positioned in a supine position with the mouth open and the jaw pulled ventrally with the help of a bandage, in such a way to better expose the defect (Figure 2A). The procedure started after all residual food had been removed and cleaned with chlorhexidine (Periovet®). A wide U-shaped incision through the entire palatine mucosa – with both greater palatine arteries present – was performed in an attempt to close the existing oronasal communication (Figure 2B). As the flap dimensions were not big enough to cover the whole defect, a synthetic (polypropylene - Marlex®) mesh was chosen and placed under the flap and subsequently sutured in a simple interrupted nylon 3-0 suture pattern at the margin of the permanent mucosa adjacent to the teeth, and kept partially exposed on the right side, just enough to cover the oronasal communication completely (Figure 2C). As there was no intolerance on the part of the animal, and good results were seen in the first postoperative period, the same previously used medications were prescribed after this new intervention. The fragment was sent for histopathology, which confirmed the suspected diagnosis of productive osteosarcoma.

The animal returned after 14 days for reevaluation and removal of the stitches. In order not to compromise the incorporation of the mesh to the oral mucosa, the mesh was maintained for another 10 days, as it had shown a slight movement when handled (Figure 2D). After the recommended period, the flap had adhered properly, with the presence of granulation tissue through the mesh orifices on the donor site (Figure 2E). After that, the animal was anesthetized again with propofol at an effective dose to remove the stitches. At this point, the animal was not taking any medication.

Six months after the second surgical procedure, the owner reported a foul smell coming from the dog’s mouth. When the owner managed to open the mouth of the animal, she noticed some whitish tissue. Also, according to her, the dog had rare sneezing episodes. During the physical examination, the animal was found to be generally well and had even gained weight. No nasal discharges or other clinical changes were seen. Cervical and prescapular lymph nodes looked normal. On the oral cavity examination, the palatal mucosal flap, as well as the polypropylene mesh, were found to be properly in place with almost complete incorporation of the mesh to the mucosa. The presence of an orifice was also seen where the third premolar should be, protected by the mesh, that was preventing the passage of any material through the nasal cavity (Figure 2F).

A new visit occurred ten months after the last procedure, as the owner had reported that the mesh placed on the palate was retaining food. She also complained that the animal had been feeling pain on its leg during the past month and reported claudication and swelling in the proximal portion of the left pelvic limb. The animal had not been eating well, had been vomiting and had not been able to walk for the past two days. After the examination, the animal was found to have lost weight. The animal was referred for chest and pelvis X-rays, which showed small widespread circular radiopaque focuses with various sizes, suggesting pulmonary metastasis, as well as a fracture on the left femoral neck, most probably of pathological origin. Fifteen days after the last visit, the dog passed away due to osteosarcoma-related complications.

## Discussion

The current case describes the use of a transposition U-shaped palatal flap associated with a synthetic polypropylene mesh to repair a defect in the transition area between the maxilla and palatine bones, after a dehiscence in the central left hemimaxillectomy surgery stitches, which met the previously established goal to heal the animal’s oronasal communication, keeping food remains and saliva from being sucked in and causing changes in the respiratory system. Additionally, although very little food retention was seen and halitosis was reported when the animal would open its mouth, the mesh was incorporated in the tissue, there was good acceptance, and the animal gained weight during approximately six months of follow-up.

A definite tumor diagnosis requires histopathological evaluation of the fragment collected in a biopsy or surgical excision (Albertus, 2011; Fossum et al., 2021; Nelson & Couto, 2023). As it is extremely variable in its histological appearance, a factor that is used for a definite diagnosis of the neoplasm is the production of osteoid by malignant mesenchymal cells (Thompson & Dittmer, 2017). In microscopy, the samples analyzed in H&E revealed a proliferation of mesenchymal origin with solid areas which, at times, would show beams displaying moderate multifocal atypical osteoid differentiation.

The surgical planning for this case was based only on the surgeon’s knowledge of reconstructive techniques, as well as the skull X-rays. As a result of this test, a radiolucent area was found close to the premolars, which were surrounded by a mass that was suggestive of bone resorption. As an alternative, according to Ghirelli et al. (2013), a computed tomography can support the pre-surgical analysis and is an essential tool for the preoperative evaluation of animals with oral formations, both maxillary and mandibular, as its greater sensitivity to differences in density, absence of overlaps and anatomical detailing, allows a more precise evaluation of any compromised bones and local invasion, thus determining tumor extension, prognosis, and the feasibility of the surgical treatment. Even though this test had been suggested to the owners, they were not interested in doing it, due to their travel distance to the closest advanced diagnostic imaging site.

Still on the benefits of and support from computed tomography for surgical planning, Kim et al. (2018) reported a surgical reconstruction case on a 12-year-old mix breed female dog with a left caudal maxillary mass. A TC had been previously done, and the images served as the basis for customizing their own 3D-printed substitute material for later replacement of the site after tumor resection. During an eight-month follow-up, the outcome was successful, and no problems were seen during the periodic TC evaluations and oral examination. Although the authors were successful in creating the prosthesis, it should be noted that the cost of the images and the material was not considered. Of note, the technique used for this case is not only a low-cost alternative, but also effective in achieving the purpose of resolving the oronasal communication.

Even though no blood transfusion was needed in this case – only food supplementation – the literature clearly indicates that hemorrhage is the most common intra-operative complication for the different descriptions and variations of the maxillectomy technique (Arzi et al., 2020). A study by Carroll and Mathews (2020) describes a modified approach to the caudal maxillectomy in dogs involving pre-ligation of the maxillary artery in an attempt to limit intra- and postoperative hemorrhage. The authors retrospectively evaluated this modified technique in 22 dogs and found that all animals that did not have the maxillary artery ligated prior to the surgery had intra-operative hypotension. Additionally, 67% of these dogs needed blood transfusion during or after the procedure, while only one animal among those that were not ligated had hypotension and only 3 required intra-operative blood transfusion, which shows the effectiveness of the technique. Still in this same study, the authors also mention facial and ocular edemas, suture dehiscence and lip ulcer as postoperative complications. The latter was similar to what happened to the upper left lip of the animal due to a lip drop caused by the absence of the upper canine. Furthermore, sneezing and choking after the procedure was reported by the owner, which are believed to have occurred due to the presence of clots in the sinus area, as well as constant contact of the mesh with sensitive receptors in the same area.

According to Guzu et al. (2021), flaps are closed in a simple interrupted pattern using 5-0, 4-0, or 3-0 absorbable monofilament suture materials such as poliglecaprone 25. For Parell and Becker (2003), and Rosenzweig et al. (2010), the comparison of the suture pattern using absorbable and non-absorbable sutures in reconstructive plastic surgery on the human face did not show a statistically significant difference in the complication of the surgical wound (infection, hematoma and dehiscence). A non-absorbable (nylon) suture was used in this report because a long-lasting absorbable material (such as poliglecaprone or polydioxanone) was not available at the time of the procedure. We believe that the presence of an orifice where the premolar should be, protected by the mesh, as seen after the six-month follow-up period, is not related to the type of suture used, but rather to other factors such as individual healing process, bacteria known to be present in the oral cavity, type of food, and constant movement of the patient’s tongue in the region.

Factors supporting suture dehiscence of surgical wounds in the oral cavity include severe bacterial contamination, inappropriate flap preparation, the passage of food through the mouth, and constant movement of the tongue (Beckman, 2006; Smith, 2001; van de Wetering, 2005). Taney et al. (2021) add that the presence of neoplasm during surgery, the area of the hard palate mucoperiosteal flap, and the distance that the apex of the flap needs to cover are associated to greater flap failure. Although the previously prepared flap was meant to occlude all oronasal communication, it had to be combined with a synthetic material to meet its purpose. After six months of evaluation, part of the flap was found to show a small dehiscence that did not harm the animal’s health in terms of local food aspiration, especially due to the presence of the polypropylene mesh under the prepared flap.

Metastasis is common in 90% of the osteosarcoma cases. Patient death commonly occurs due to metastatic diseases in the lung, mostly affecting the pulmonary parenchyma in patients with osteosarcoma in the appendicular skeleton (Albertus, 2011; Straw, 2004). However, it has been described that the metastatic potential of axial osteosarcomas in dogs varies according to its location, even though those in the mandible and maxilla tend to be considered as having a low metastatic potential compared to other locations of the tumor in the axial skeleton (Farcas et al., 2014). It cannot be said that the spontaneous fracture the patient developed in the left femoral neck region after almost 10 months is a metastasis, as no cytological or histopathological analyses were performed on the site, even though pathological fractures are known to be common in patients with bone neoplasms or bone diseases caused by nutritional and hormonal imbalance.

Differences in behavior are associated with the location of the tumor (Farcas et al., 2014). In a study by Wallace et al. (1992), the authors evaluated 69 dogs undergoing hemimaxillectomy to treat oral tumors and concluded that in surgically treated dogs with maxillary osteosarcoma – with a 1-cm surgical margin based on the clinical and radiographic evaluations – the average survival time was 4.6 months, with a 17% survival in one year. Another similar study reported that the average survival of animals is related to the location of the tumor inside the oral cavity, instead of its type. The average survival was 30 months for rostral tumors, 8 months for central tumors, and 5 months for caudal tumors. The authors reported that these results are probably related to the ability of the surgeon to achieve complete resection of the tumor (Schwarz et al.,1991). This dog’s survival was 10 months before the emergence of signs in the pelvis, indicating that the central left hemimaxillectomy procedure was properly done according to the literature, as well as the histopathological result – with uncompromised surgical margins.

Alternatives in the attempt to solve congenital or acquired defects in the oral cavity include mucosal flaps (Guzu et al., 2021; Taney et al., 2021) or the use of biomaterials (Castejón-González & Reiter, 2023; Edstrom & Smith, 2014). Although the materials that were used meet the initial purpose and have a good aesthetic appearance, it is uncommon to conduct histological analyses that reveal the material-body interaction in the short, mid, and long term. Similarly, histological analyses were not done for this study, even though there was an opportunity to do so during the subsequent evaluation periods, which could have brought more light into the use of the mesh for at least six months. Thus, we suggest that this evaluation can be done in further research to bring additional information on the interaction of this material and the body.

## Conclusion

The use of the transposition U-shaped palatal flap combined with a polypropylene mesh to correct oronasal communication in a dog is a technically easy, low-cost and efficient option for the proposed treatment.
